# Supplementary material for: Optimizing CT Esophagography: Ex Vivo Study on Contrast Ratios, Image Quality, and Dual-Energy Benefits
Source: Bioengineering (Basel). 2024 Dec 20;11(12):1300. doi: 10.3390/bioengineering11121300 (PMC11727102; doi:10.3390/bioengineering11121300)
Supplement: Supplementary file 1 [file bioengineering-11-01300-s001.zip › bioengineering-3271980-supplementary.pdf]

Supplementary Material S1

Detailed information about the phantom experiment

Oral contrasts were prepared such that four iodine contrast agents (320 mgI/mL, 350 mgI/mL, 370 mgI/mL, and 400 mgI/mL) were diluted by normal saline in 21 volume ratios(1:0, 1:1, 1:2, 1:3, 1:4, 1:5, 1:6, 1:7, 1:8, 1:9, 1:10, 1:11, 1:12, 1:14, 1:15, 1:17, 1:19, 1:24, 1:49, 1:99, and 1:199). Eppendorf tubes (5mL) filled with the prepared oral contrasts of different dilution ratios were numbered and placed sequentially in hollow plastic tubes (inside diameter: 20 mm). Then, the hollow plastic tube was positioned in a plastic box (length: 450 mm, width: 300 mm, height: 160 mm) filled with water with its long axis perpendicular to the scan plane (Figure 1). The reason that tubes were not placed side by side was to avoid the interaction of BHAs and noise generated by tubes filled with contrast medium. Conventional helical CT scans under three tube voltages (80 kVp, 100 kVp, and 120 kVp) were performed on the phantom. Automatic tube current (range: 50-150 mA) was controlled at a noise index of 12. Images were reconstructed at a thickness of 1.25 mm by ASIR 40% for image quality evaluation.

To further evaluate the additional value of dual-energy CT for the improvement of image quality, the phantom with iodine contrast agent (370 mgI/mL) underwent a dual-energy scan (Revolution CT, GE HealthCare). The scanning parameters are summarized as follows: fast tube voltage switching between 80 and 140 kVp; tube current: 200 mA, pitch: 0.992; reconstruction thickness: 1.25 mm; slice interval: 1.25 mm. Monochromatic images with or without MARs on (40, 60, 70, 80, 90, 100, 120, and 140 kiloelectron volts [keV]), 100 kVp-like and 120 kVp-like images, and virtual unenhanced images were additionally obtained. The image quality was compared with images acquired from conventional helical series by the two radiologists.

Table S1. Details of oral contrast agents in the literature for CT esophagography.

Reference	Contrast Agent Stock Solution	Dilution Ratio	Diluted Concentration (mg I/mL)	Applicable Scenario
Norton-Gregory et al., 2021 [6]	350 (Iohexol, Omnipaque)	1:0	350	Emergent esophageal rupture or postoperative leak
Lantos et al., 2013 [16]	370 (Gastrografin, Bristol-Myers Squibb)	1:0	370	Postoperative leaks after esophagectomy
Evans et al., 2024 [19]	367 (MD-Gastroview, Guerbet)	1:0	367	Suspected esophageal perforation
Wei et al., 2020 [2]	370 (Gastrografin, bracco)	1:0	370	Suspected esophageal perforation
Conradie et al., 2015 [15]	300 (Iohexol, Omnipaque)	1:1	150	Penetrating upper digestive tract injuries
Strauss et al., 2010 [13]	300 (Iohexol, Omnipaque)	1:5	50	Intrathoracic anastomotic leak complicating esophagogastrectomy
Palacio et al., 2018 [14]	NA (Iohexol, Omnipaque)	1:7	NA	Anastomotic leaks in post-esophagectomy care
Tonolini et al., 2013 [9]	NA (NA)	1:9	NA	Suspected boerhaave syndrome
Fadoo et al., 2004 [7]	300 (Iohexol, Omnipaque)	1:9	30	Esophageal perforation and other causes of acute chest pain
Suarez-Poveda et al., 2014 [10]	300 (Iobitridol, Omnipaque)	1:9	30	Esophageal rupture, especially in trauma cases or other patients with clinical suspicion
Masarapu et al., 2020 [18]	NA (NA)	1:9	NA	Acute chest pain caused by emergent esophageal conditions
Saksobhavivat et al., 2016 [20]	300 (NA)	1:19	15	Gastrointestinal injury after penetrating torso trauma
Hogan et al., 2008 [11]	370 (Gastrografin)	1:49	7.4	Anastomotic leak following oesophagogastric surgery
Terrazas et al., 2020 [21]	300 (Iopamidol, Isovue)	1:99	3	Esophageal injuries
Upponi et al., 2008 [12]	300 (Iopamidol, Bracco)	1:150	1.98	Occult anastomotic leaks following oesophagectomy
Little et al., 2020 [17]	NA (NA)	NA	NA	Esophago-airway fistula

Table S2. Intra-reader agreement of objective assessment.

	ROIa		ROIb		ROIc		ROId	
	**mean-HU**	**SD**	**mean-HU**	**SD**	**mean-HU**	**SD**	**mean-HU**	**SD**
ICC	0.995	0.979	0.917	0.996	0.835	0.996	0.872	0.901
P value	<0.001	<0.001	<0.001	<0.001	<0.001	<0.001	<0.001	<0.001
